# Improvement of High Affinity and Selectivity on Biosensors Using Genetically Engineered Phage by Binding Isotherm Screening

**DOI:** 10.3390/v11030248

**Published:** 2019-03-12

**Authors:** Jong-Min Lee, Eun Jung Choi, Juyun Park, Vasanthan Devaraj, ChunTae Kim, Jiye Han, Won-Geun Kim, Kyujung Kim, Yong-Cheol Kang, Kwang Ho Kim, Jin-Woo Oh

**Affiliations:** 1 Research Center for Energy Convergence and Technology, Pusan National University, Busan 46241, Korea; jongminlee1984@gmail.com (J.-M.L.); choi1203@hanmail.net (E.J.C.); devarajvasanthan@gmail.com (V.D.); 2 Department of Chemistry, Pukyong National University, Busan 48513, Korea; sai3162@naver.com (J.P.); yckang65@gmail.com (Y.-C.K.); 3 Department of Nano Fusion Technology, Pusan National University, Busan 46241, Korea; chuntae1122@gmail.com (C.K.); hanyksw20@naver.com (J.H.); kim1guen@gmail.com (W.-G.K.); 4 Department of Optics and Mechatronics Engineering, Pusan National University, Busan 46241, Korea; kjkimgo@gmail.com; 5 School of Materials Science and Engineering, Pusan National University, Busan 46241, Korea; 6 Department of Nanoenergy Engineering, Pusan National University, Busan 46241, Korea

**Keywords:** M13 bacteriophage, directed evolution, binding affinity, surface-enhanced Raman scattering

## Abstract

The genetically engineered M13 bacteriophage (M13 phage), developed via directed evolutionary screening process, can improve the sensitivity of sensors because of its selective binding to a target material. Herein, we propose a screening method to develop a selective and sensitive bioreporter for toxic material based on genetically engineered M13 phage. The paraquat (PQ)-binding M13 phage, developed by directed evolution, was used. The binding affinities of the PQ-binding M13 phage to PQ and similar molecules were analyzed using isothermal titration calorimetry (ITC). Based on the isotherms measured by ITC, binding affinities were calculated using the one-site binding model. The binding affinity was 5.161 × 10^−7^ for PQ, and 3.043 × 10^−7^ for diquat (DQ). The isotherm and raw ITC data show that the PQ-binding M13 phage does not selectively bind to difenzoquat (DIF). The phage biofilter experiment confirmed the ability of PQ-binding M13 bacteriophage to bind PQ. The surface-enhanced Raman scattering (SERS) platform based on the bioreporter, PQ-binding M13 phage, exhibited 3.7 times the signal intensity as compared with the wild-type-M13-phage-coated platform.

## 1. Introduction

Recently, there have been numerous studies about biosensors to detect various meaningful molecules. Some approaches have focused upon developing and improving the sensing systems, while others have been trying to enhance the sensitivity using interesting sample preparation, such as nanostructures [1,2]. For example, surface-enhanced Raman scattering (SERS) sensors are utilized in the biological [3,4], environmental [5], and chemical [6] fields, due to their extremely high detection sensitivities, even at the single molecular level [7,8,9,10,11,12]. The main mechanism of SERS is electromagnetic field enhancement, which originates from the plasmon resonance with emission process and Raman excitation. Through this mechanism, an active control over surface plasmon resonance outputs tunable SERS signals, which can be applied to on-demand detection of biomolecules and chemicals. It is known that introduction of a functional reporter to SERS platform improves sensitivity and provides selectivity [13,14,15,16]. When the functional reporter is coated on a SERS sensor, the detection sensitivity increases, because the probability of entrapment of the target substance in a hotspot increases.

Even though these approaches provide significant solutions for detecting extremely tiny molecules, we should note that surface modification with high affinity and selectivity with targets may be a key factor in designing a biosensor. Thus, there have been many biological and chemical methods introduced to obtain high affinity with specific targets [17,18]. Among the approaches, George Smith, the winner of the 2018 Nobel Prize in Chemistry, developed an amazing method, known as bacteriophage display, as an alternative for antibodies in 1995 [19,20]. The M13 bacteriophage (M13 phage) is a functional bionanowire with a length of 880 nm and a diameter of 6.6 nm [21]. Unlike chemical synthesis methods, it is possible to precisely replicate the same objects at once because the DNA information assembles them. Using this method, M13 phage that selectively binds to a target material can be developed. Due to the well-defined geometry and ability to retain diverse functional peptides through directed evolution, it has been applied to various fields, such as optical sensors [22,23], scaffolds [24,25,26], energy devices [27,28,29,30], cancer therapy [31] and drug-delivery systems [32]. Although phage display is a suitable method to find the best peptide sequence for a target material, the binding affinity between one peptide and several targets should be verified by using a different method. To improve the SERS sensor using M13 phage as a functional bioreporter, an evaluation of the binding affinity of the M13 phage to various targets is necessary.

Paraquat (*N*,*N*’-dimethyl-4,4’-bipyridinium dichloride) and diquat (1,1’-ethylene-2, 2’-bipyridinium dibromide) are bipyridylium herbicides that are used as chemical agents. They are immediately absorbed into the body and distributed to the lungs and kidneys, along with the bloodstream [33]. These dangerous substances lead to death by disabling the function of each of the organs of the human body. The toxic mechanism is the result of redox circulation, such as through cellular NADPH depletion and peroxide radicals, which produces toxic oxygen [34]. Generally, pesticide residue can be analyzed by liquid chromatography (LC) or a UV-detector method with the sensitivities at 0.05 ppm level. In 1986, FAO/WHO JMPR suggested a maximum daily intake of 0.004 (daily intake allowance, ADI), so high-sensitivity sensor technology is required [35]. Moreover, it is necessary to develop a technology to analyze the pesticide residues in situ and real time. Based on functional peptides that specifically bind to bipyridylium pesticides, we have developed a phage-bioreporter material through genetic engineering that can be used in biosensors. Using the functional bacteriophage-bioreporter material, we applied to the SERS-based in situ and real-time analysis sensor platform and achieved 0.01 ppm-level detection limits.

**Scheme 1:**
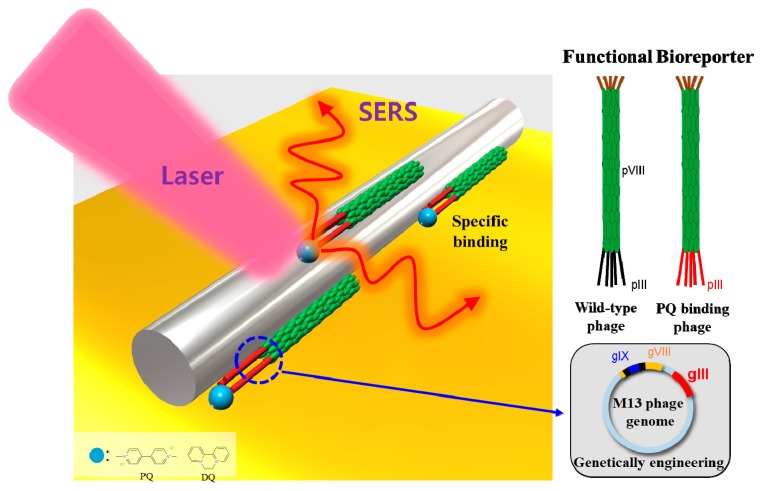
The schematic diagram of genetically engineered M13 bacteriophage as a bioreporter and its application for a surface-enhanced Raman scattering (SERS) platform. The genetically engineered M13 bacteriophage was decorated on the silver nanowire surface.

In this work, we provide a screening method to develop sensitive and selective M13 phage-based bioreporter for toxic material detection. Paraquat (PQ; a pesticide) was used as the target substance because of its toxicity in man and animals [36]. In order to confirm the selectivity, diquat dibromide monohydrate (DQ) and difenzoquat methyl sulfate (DIF) were also used, which are similar in molecular structure to PQ. M13 phage topically expressing a peptide that selectively binds PQ was developed by genetic manipulation, and the binding of target materials by this peptide was determined by isothermal titration calorimetry (ITC). X-ray photoelectron spectroscopy (XPS) was used to analyze a phage biofilter structure to confirm the binding between genetically engineered M13 phage and PQ. SERS experiments were performed to confirm that the bioreporter contributes to sensors using genetically different types of M13 phage for the target substance, PQ, and were also performed using the PQ-binding phage for the target substances PQ, DQ, and DIF. As illustrated schematically in Scheme 1, the genetically engineered M13 bacteriophage can act as a reporter and bind the target substance around the hotspot. On the same SERS platform, the number of target substances captured in the hotspot determines the SERS intensity. Therefore, the SERS platform, with the genetically engineered M13 phage, can obtain a larger SERS signal as compared with the SERS substrate with the wild-type M13 phage that cannot act as a reporter.

## 2. Materials and Methods

### 2.1. Materials

The PQ-binding peptide (PQB-P, sequence: Ser (S)–Pro (P)–Pro (P)–Trp (W)–Pro (P)–Pro (P)–Arg (R)–Pro (P)) and WHW peptide (WHW-P, sequence: Trp (W)–His (H)–Trp (W)) were synthesized in Bionics (Seoul, Korea). Methyl viologen dichloride hydrate (PQ), diquat dibromide monohydrate (DQ), difenzoquat methyl sulfate (DIF), and thiophenol (benzenethiol, BT) were purchased from Sigma-Aldrich (St. Louis, MO, USA). The PQ-binding M13 bacteriophage (PQB-BP) and the WHW-type M13 bacteriophage (WHW-BP) were genetically engineered and mass-cultured.

### 2.2. Genetic Engineering

To incorporate PQB-P, we genetically engineered the minor protein of M13 phage (pIII). The minor coat proteins of M13 bacteriophage were modified with SPPWPPRP by site-directed mutagenesis PCR. We designed the partial target sequence SPPWPPRP using the primer 5′-CTATTCTCACTCGCCGCCCTGGCCACCCCGGCCGGCCGAAACTG-3′ and the reverse primer 5′-CAGTTTCGGCCGGCCGGGGTGGCCAGGGCGGCGAGTGAGA-ATAG-3′ to construct a linear insertion peptide complementary to the engineered gIII 3′–5′ region. To incorporate gene sequences, PCR amplification was performed using Pfu DNA Polymerase, two primers (insertion and linearization) and an M13KE (NEB, Ipswich, MA, USA) vector as a template. The obtained product was incubated with *Dpn*1 and transformed into XL1-Blue electroporation competent bacteria. The DNA sequencing facility (Bionics Inc., Seoul, Korea) verified the amplified plasmid sequence (Figure S7 in Supplementary Information). Constructed phages were amplified using bacterial cultures and purified by polyethylene glycol precipitation. The phage stability was confirmed by verifying the DNA sequences at each step of the amplification.

### 2.3. Fabrication of Phage Biofilter

A sufficiently thick phage biofilter was required for the XPS study to avoid generating strong signals from the silicon substrate. To fabricate the phage biofilter, we used a 0.5 cm × 0.5 cm silicon substrate which had been surface-treated with oxygen (O_2_) plasma to improve hydrophilicity. Concentrations of phages (PQB-BP and wild type) and poly(diallyldimethylammonium chloride) (PDDA) solutions were 3 mg/mL and 2 wt %, respectively. In order to improve the adhesion between M13 phage and the substrate, 50 μL of PDDA was applied and naturally dried. M13 phage (50 μL) was also coated in the same way [37].

### 2.4. Preparation of SERS Sensor Platform Functionalized by M13 Phage

To evaluate the sensitivities with PQB-BP or wild type, a silver nanowire on gold film (SNGF) SERS sensor platform was used for the bare SERS sensor platform [38]. The advantage of this platform is that it allows measurement of a single nanowire per laser spot because nanowires were visible under an optical microscope. The gold substrate was fabricated by depositing 100 nm gold on a silicon substrate (<100>, p-type) using a thermal evaporator. Silver nanowires (NWs) were fabricated using a conventional solution method using polyvinylpyrrolidone (PVP) as a stabilizing agent and coating. The diameter of the silver NW was ~300 nm and its length was ~30 μm. Silver NW solution (20 μL) was applied to a gold-coated substrate of 0.5 cm × 0.5 cm, which was then naturally dried. The solution was diluted until a single nanowire on the substrate was visible by optical microscopy. The concentration of PQB-BP was 0.1 μg/mL, and 20 μL was added to the bare SERS sensor platform.

### 2.5. ITC Measurement

ITC directly measures and analyzes exothermic and endothermic reactions at the molecular level and, in the present study, binding isotherms were obtained using the Affinity ITC system (TA Instruments, New Castle, DE, USA). Since ITC measures a slight temperature change of even 0.1 μJ, microbubbles need to be removed from the solutions to be used. Microbubbles of PQB-P solution were removed when it was stored in a vacuum for ~10 min. Since pesticides are volatile, they were centrifuged at 135,000 rpm for 10 min without using a vacuum. The volume of the cell used was 1300 μL, and samples (10 μL) were injected at 0.5 μL/s every 300 s using a syringe. The binding isotherms were calculated using the TA instrument’s dedicated software. The fitting model used for the calculation is the one-site binding model.

### 2.6. Analysis of Adsorbed Ion on Phage Biofilter Structures

The phage biofilter structures were analyzed by XPS (ESCALAB 250, Thermo Fisher Scientific, Waltham, MA, USA), equipped with a hemispherical analyzer and a twin anode non-monochromatic Al Kα source. To evaluate the binding ability of PQB-BP, phage biofilters made of PQB-BP and wild type were immersed in 1 ppm PQ solution three times for a second, and allowed to air-dry at room temperature. Selective chloride ion filtration was demonstrated when the PQ molecule was extracted with phage biofilter.

### 2.7. SERS Measurement

Raman spectra were obtained using a high-resolution Raman/photoluminescence spectrophotometer (LabRAM HR-800 UV-Visible-NIR, Horiba Jobin Yvon, Kyoto, Japan). The excitation wavelength used was 633 nm, and the laser power was set at 10 μW. The measurement time was 10 s (Figures S1 and S2 in Supplementary Information).

## 3. Results and Discussion

### 3.1. Binding Characteristics of the PQ-Binding Peptide

Hua and his colleagues developed the PQ-binding peptide using phage display and verified the binding affinity in cell experiments [39]. However, this result did not guarantee that binding would occur in other environments. Binding isotherms obtained by ITC provide a direct means of measuring binding affinity. Accordingly, we measured the binding constant of PQB-P with PQ, DQ, and DIF. The binding affinity of WHW-P to PQ was analyzed for reference purposes. WHW-P has been widely utilized as a good bioreporter for aromatic molecules [16,40].

**Figure 1:**
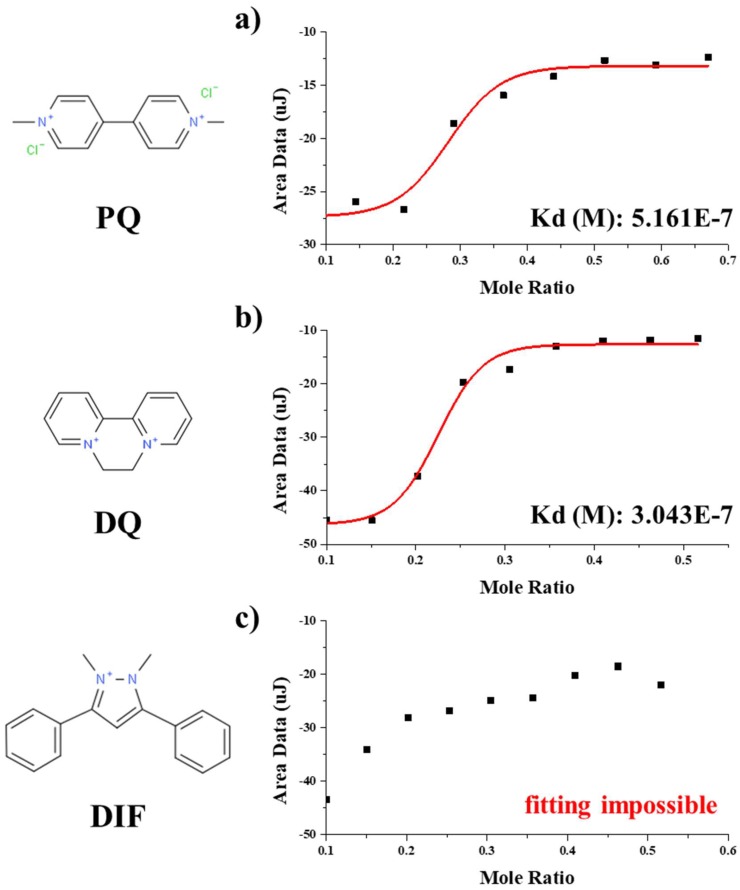
Binding isotherm of the PQ-binding phage to (**a**) paraquat (PQ), (**b**) diquat (DQ), and (**c**) difenzoquat (DIF) measured by ITC. Red line is the result of fitting using the one-site binding model. In the DIF result, fitting is impossible because the raw ITC data is not stable (Figure S6 in Supplementary Information).

Figure 1a shows the binding isotherm of PQB-P with PQ. PQB-P (0.8 mM) diluted in DI water was added to the syringe, and 0.12 mM PQ diluted in DI water was added to the cell. Raw ITC data for PQB-P to PQ shows that an exothermic reaction was stably displayed for each injection (Figure S3 in Supplementary Information). This result means that the PQ molecule was stably adsorbed onto PQB-P. The binding constant for PQB-P to PQ was ~5 × 10^−7^, which is better than the binding constant of WHW-P to trinitrotoluene (TNT) [40]. The exothermic reaction of PQB-P to PQ was saturated before the molar ratio is halved. This is because many PQ molecules were stably adsorbed to one PQB-P. Also, since PQB-P and PQ are firmly attached, no further binding occurred after saturation. Figure S4, in the Supporting Information, presents raw ITC data for WHW-P to PQ. Unlike PQB-P, raw ITC data for WHW-P to PQ shows that endothermic and exothermic reactions were repeated for each injection. This result indicates that the PQ molecule was not stably adsorbed on WHW-P.

Figure 1b,c show binding isotherms for PQB-P to DQ or DIF. 0.28 mM DQ diluted in DI water. Since DIF is insoluble in water, it was first diluted to 1000 ppm in methyl- alcohol and diluted with DI water to a concentration of 0.28 mM. Other measurement conditions were the same as those using PQB-P and PQ. The binding constant for PQB-P to DQ was ~ 3 × 10^−7^ using the same model as PQ (Figure S5 in Supplementary Information). The binding isotherm for PQB-P to DIF was entirely different from the others, and the one-site binding model did not work. Raw ITC data for PQB-P to DIF (Figure S6 in Supplementary Information) showed unstable behavior similar to that observed for WHW peptide to PQ.

Even though the molecular structures of PQ, DQ, and DIF are similar, PQ and DQ are pyridine-based compounds, and DIF is pyrazolium-based compound. There is one tryptophan for SPPWPPRP, which is a functional peptide of PQB-BP. We can assume that a tryptophan ring was bound to the pyridine ring of the localized aromatic site with π- π interaction. However, considering that the tryptophan rings of WHW peptide don’t contribute efficiently to bind pyridine ring, it was expected that more complicated actions were present.

### 3.2. Phage Biofilter Experiments

A phage biofilter experiment was performed to investigate the binding ability of M13 phage expressing PQB-P. Even though the DNA sequence of PQB-BP was verified, the phage biofilter experiment using an actual M13 bacteriophage has significance for duplication confirmation. This experiment was confirmed to be effective for extracting ions in solution [37]. In this study, the method of producing the phage biofilter was simplified as the simple structure matches the purpose of comparing PQB-BP and wild type.

**Figure 2:**
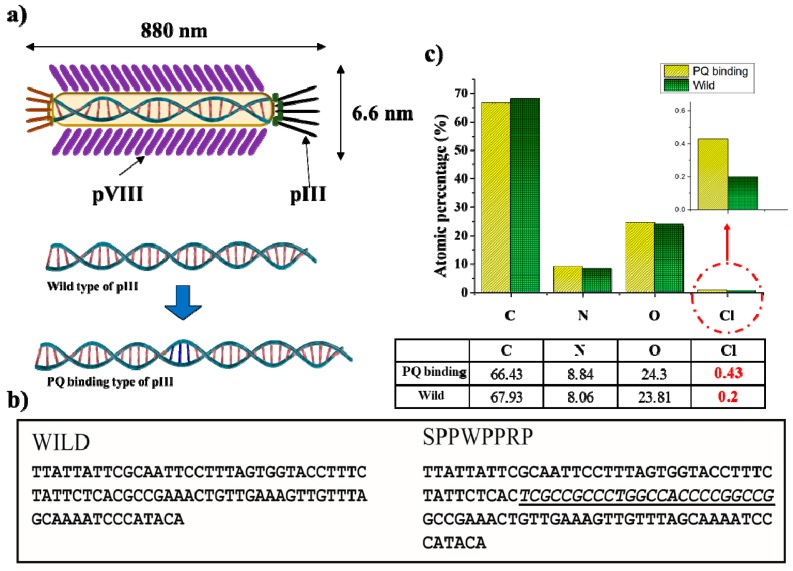
(**a**) Structure of M13 phage. (**b**) pIII genetic engineering site information shows conversion from wild type to SPPWPPRP-type phage (the PQ-binding phage). (**c**) Atomic percentages of biofilter structure measured by XPS.

Since the XPS signals of the substrate interfere with other signals from the sample surface, they must be suppressed as much as possible. The XPS signals from the silicon substrate were suppressed, which confirmed that the fabricated phage biofilter was sufficiently thick (Figure S2 in Supplementary Information). Figure 2c shows the relative atomic ratios of the two types of phage films. Relative atomic ratios of carbon, nitrogen, and oxygen were similar for the two biofilters. This means that two phage biofilters were well produced under the same conditions. However, the relative atomic ratio of chlorine ions measured by the biofilter made of PQB-BP was more than twice that observed for the biofilter made of wild type. This result indicates that PQB-BP efficiently binds PQ compared to wild type.

### 3.3. SERS

**Figure 3:**
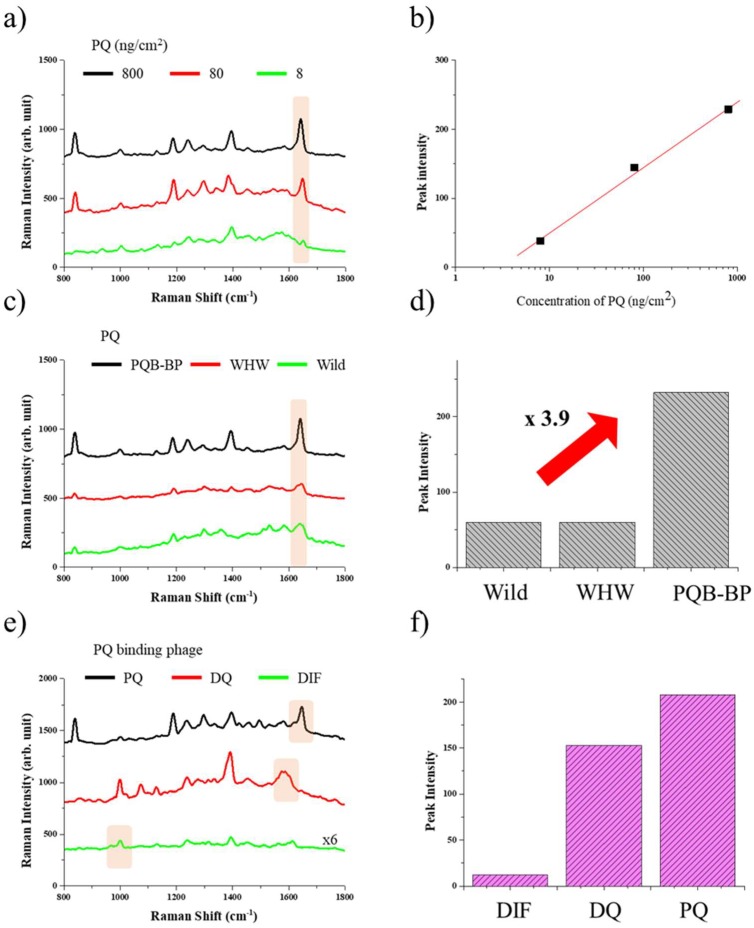
(**a**) Raman spectra of PQ on the PQB-BP-coated SERS platform. (**b**) Raman intensity comparisons of (**a**). (**c**) Raman spectra of PQ on the PQB-BP, WHW-BP, and wild-type-coated SERS platforms. (**d**) Raman intensity comparisons of (**c**). (**e**) Raman spectra of PQ, DQ, and DIF on the PQ-binding-phage-coated SERS platform. (**f**) Raman intensity comparisons of the PQB-BP-coated SERS platform for the three different pesticides.

Figure 3a shows Raman spectra obtained at various PQ concentrations (8, 80, or 800 ng/cm^2^) on the PQB-BP-coated SERS platform. The drop volume of the pesticides was fixed at 20 μL. The characteristic Raman peak of PQ is found at ~1640 cm^−1^. The SERS intensity of PQ gradually increased with increasing concentration of PQ (Figure 3b). The results obtained show that the limit of detection (LOD) for PQ of the PQB-BP-coated SERS platform was lower than 8 ng/cm^2^ [41]. Since this SERS platform is based on a single nanowire, the hotspot density is low, but it shows a good LOD. Accurate LOD analysis was not carried out because it is outside the purpose of this paper.

In the phage biofilter experiment, we found that PQB-BP could extract PQ more efficiently than wild type, indicating the sensitivity of the PQB-BP-coated SERS platform is better than the wild-type-coated SERS platform. Figure 3c shows SERS spectra of PQ for the PQB-BP-, WHW-BP-, or wild-type-coated SERS platform. The concentration of PQ was 800 ng/cm^2^. In fact, the SERS intensity of the PQB-BP-coated SERS platform was ~3.9 times greater than wild type. Genetically engineered phages for a specific target should always have better binding affinity than wild type upon the right choice of peptide surface modification by means of genetic engineering. The genetically engineered M13 bacteriophage can be mass-cultured at low cost and can be added to all types of structures.

In addition, we found that PQB-BP is superior to WHW-BP. WHW-BP contains 2700 copies of WHW-P on its surface, whereas the PQB-BP contains only 5 copies of PQB-P at the pIII of phage. However, minor proteins (pIII) of phage can manipulate longer sequences than major proteins (pVIII). As confirmed in the ITC experiments, SPPWPPRP peptide is effective for PQ, as compared to WHW peptide. On the one hand, since the minor proteins can handle longer sequences by genetic engineering, the effective peptide could be incorporated. On the other hand, monomolecular-scale target materials have been handled in many sensor experiments with M13 phage, but the target substance dealt with here now is relatively large. In order to stably bind large molecules, the long functional peptide is necessary. This result shows that when genetically engineered phage is used in a SERS platform, it is advantageous to use a phage that exhibits strong binding ability, even if the number of functional peptides expressed in phage is small.

Figure 3e shows SERS signals of PQ, DQ, or DIF measured using the PQB-BP-coated SERS platform. The concentration of PQ, DQ, or DIF were 800 ng/cm^2^. The characteristic Raman peaks of DQ and DIF were at ~1580 cm^−1^ and ~1000 cm^−1^, respectively. Figure 3f shows that SERS signal intensity increased for all three pesticides when PQB-BP was added. Similar to the binding isotherm results, SERS signals of PQ and DQ increased significantly more than that of DIF. The result indicates that the PQ-binding phage imbued the SERS platform with selectivity. By analysis of the binding isotherm of a genetically engineered M13 bacteriophage to target substances, we can predict the selectivity of the SERS platform using M13 phage. This method will be useful for developing a SERS platform for selective detection using the genetically engineered M13 phage.

## 4. Conclusions

In summary, we demonstrated that a genetically engineered M13 bacteriophage could be used to selectively functionalize a SERS-sensor platform for improved sensitivity. We measured the binding affinity of PQ-binding peptide to some pesticides using ITC. Experimental results showed that the PQ-binding peptide selectively and stably binds to paraquat and diquat, which are pyridine-based compounds, but not to difenzoquat, which is a pyrazolium-based compound. Furthermore, it was possible to conclude that the WHW peptide cannot stably bind with PQ. The phage biofilter experiment confirms the binding ability of the PQ-binding peptide expressed in M13 phage. The results indicate that a phage biofilter made of the PQ-binding phage efficiently extracts PQ compared to a phage biofilter made of wild type. The same trend as the above results is also displayed on the M13-phage-coated SERS sensor platform. The SERS platform coated with the PQ-binding phage shows a SERS intensity which is 3.9 times stronger than that coated with wild type. The confirmed selectivity with isotherm, measured by ITC, agreed well with the SERS results. The development of bacteriophage receptors using ITC screening suggests a method to functionalize the selectivity in SERS platform. Additionally, our study may contribute to sensitivity improvement for SERS sensor platforms at low cost.

## Supplementary Materials

Supplementary materials can be found at https://www.mdpi.com/1999-4915/11/3/248/s1.
