# Supplementary material for: Improvement of High Affinity and Selectivity on Biosensors Using Genetically Engineered Phage by Binding Isotherm Screening
Source: Viruses. 2019 Mar 12;11(3):248. doi: 10.3390/v11030248 (PMC6466209; doi:10.3390/v11030248)
Supplement: Supplementary file 1 [file viruses-11-00248-s001.pdf]

Supplementary Information

# Improvement of high affinity and selectivity on biosensor using genetically engineered phage by binding isotherm screening


*Jong-Min Lee[a], Eun Jung Choi[a], Juyun Park[e], Vasanthan Devaraj[a], ChunTae Kim[b], Jiye Han[b], Won-Geun Kim[b], Kyujung Kim[e], Yong-Cheol Kang[f], Kwang Ho Kim[d,*], Jin-Woo Oh[a,b,c,*]*

[a] Research Center for Energy Convergence and Technology, Pusan National University, Busan 46241, Republic of Korea
[b] Department of Nano Fusion Technology, Pusan National University, Busan 46241, Republic of Korea
[c] Department of Nanoenergy Engineering, Pusan National University, Busan 46241, Republic of Korea
[d] School of Materials Science and Engineering, Pusan National University, Busan 46241, Republic of Korea
[e] Department of Optics and Mechatronics Engineering, Pusan National University, Busan 46241, Republic of Korea
[f] Department of Chemistry, Pukyong National University, Busan 48513, Republic of Korea

*Corresponding authors:
Prof. Kwangho Kim
School of Materials Science and Engineering
Pusan National University
Busan 46241, Republic of Korea
E-mail: kwhokim@pusan.ac.kr

Prof. Jin-Woo Oh
Research Center for Energy Convergence and Technology
Department of Nano Fusion Technology
Department of Nanoenergy Technology
Pusan National University
Busan 46241, Republic of Korea
E-mail: ojw@pusan.ac.kr

**Supporting Figures**

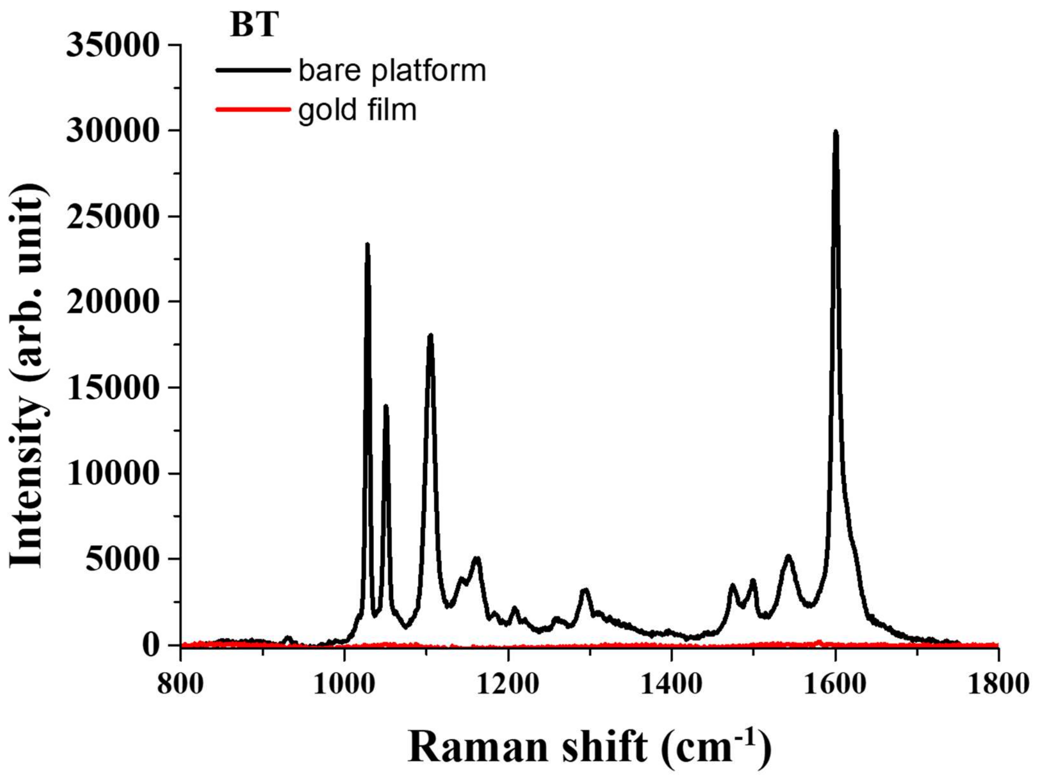


Fig. S1. Raman spectra of monolayer BT coated on the bare SERS platform (black line) and the gold film (red line). On the SERS platform, the Raman signal intensity of monolayer BT reaches over $10^4$, but it is impossible to measure in the gold film.

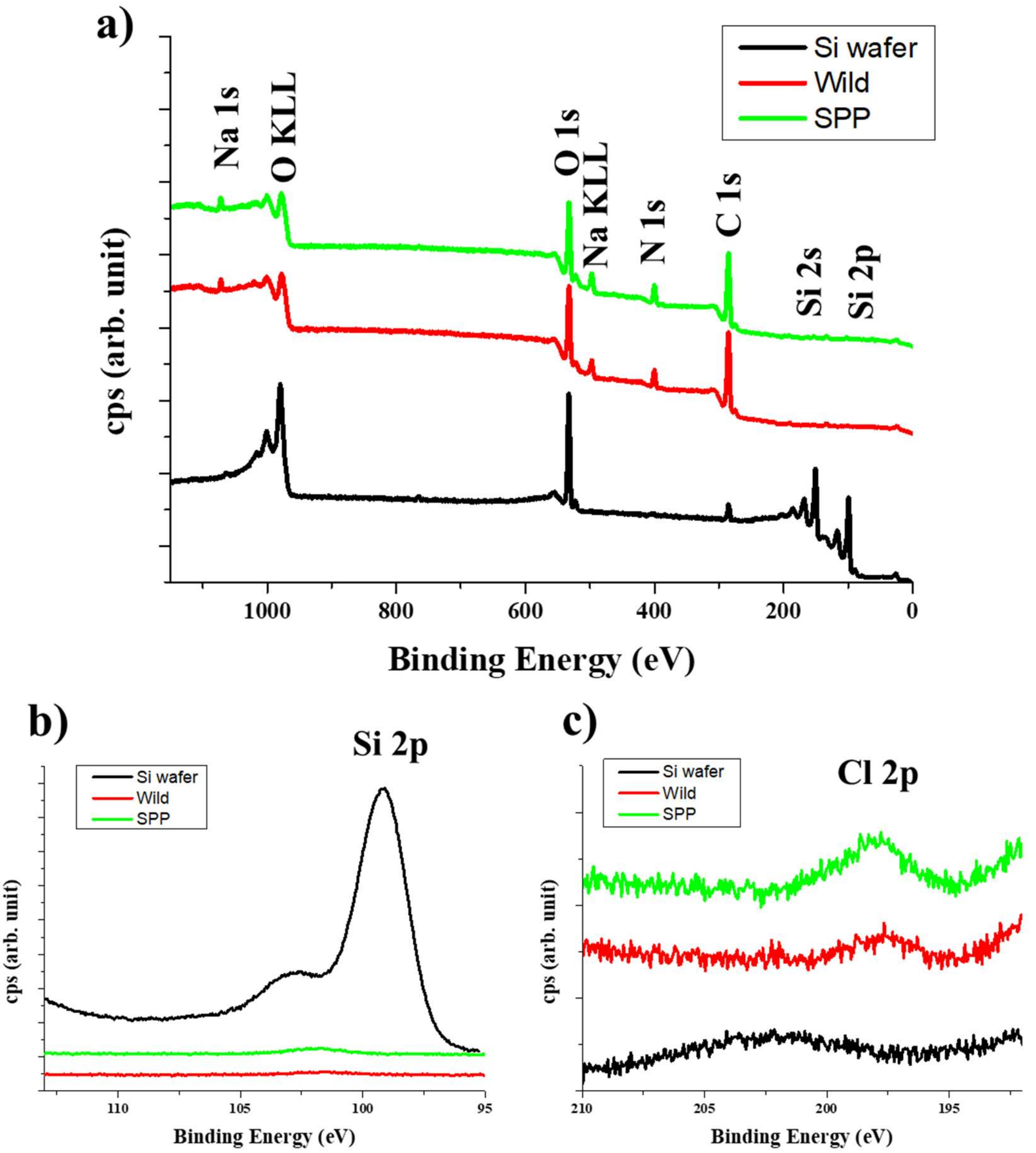


Fig. S2. XPS spectra of the phage bio-filters (the PQ binding phage and wild type) and silicon substrate. The phage bio-filters were immersed in PQ solution with 1 ppm. (a) XPS Survey spectra. (b) XPS spectra of Si 2p. Both the phage bio-filters were fabricated on Si substrates, but the Si signal do not appear in the XPS spectra. This result indicates that the phage bio-filter was made thick enough. (c) XPS spectra of Cl sp. The XPS spectrum of Si substrate to Cl 2p indicates that the Si signal does not overlap with the Cl signal.

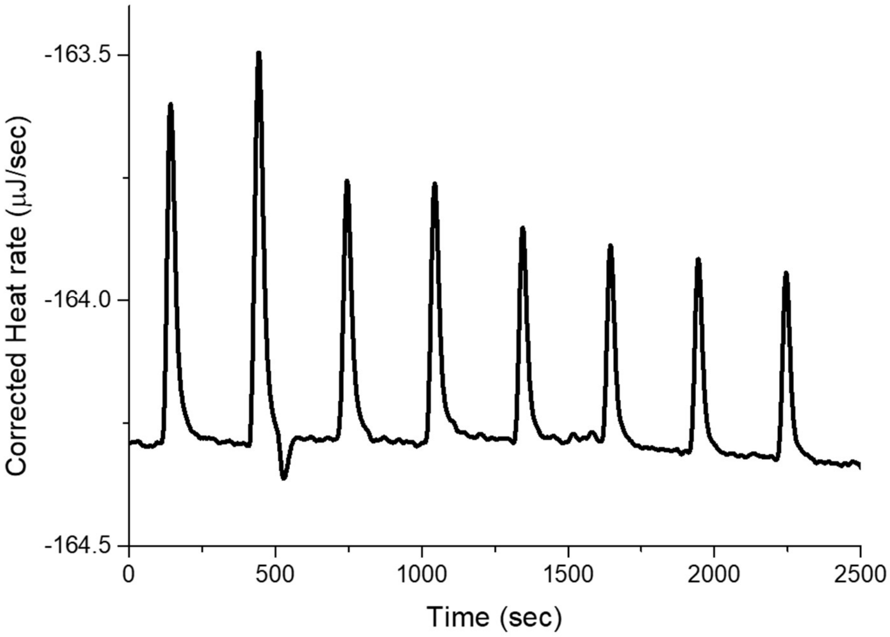


Fig. S3. Raw heat ratio of the PQ binding peptide to PQ measured by ITC.

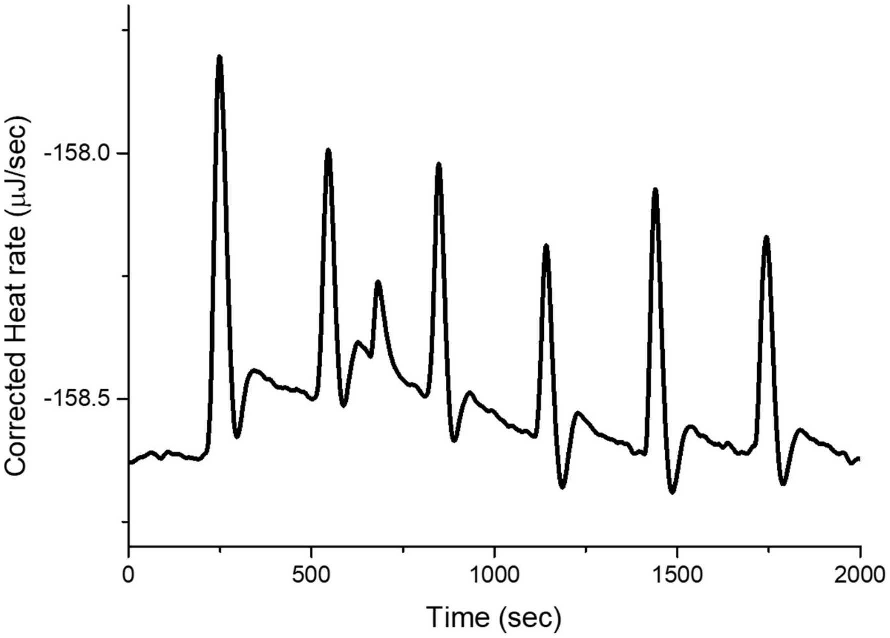


Fig. S4. Raw heat ratio of WHW peptide to PQ measured by ITC.

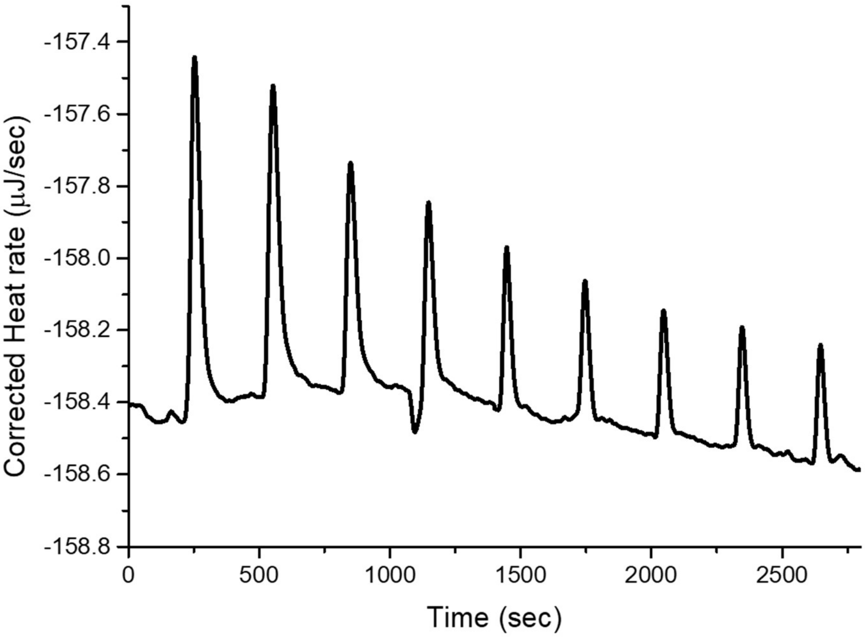


Fig. S5. Raw heat ratio of the PQ binding peptide to DQ measured by ITC.

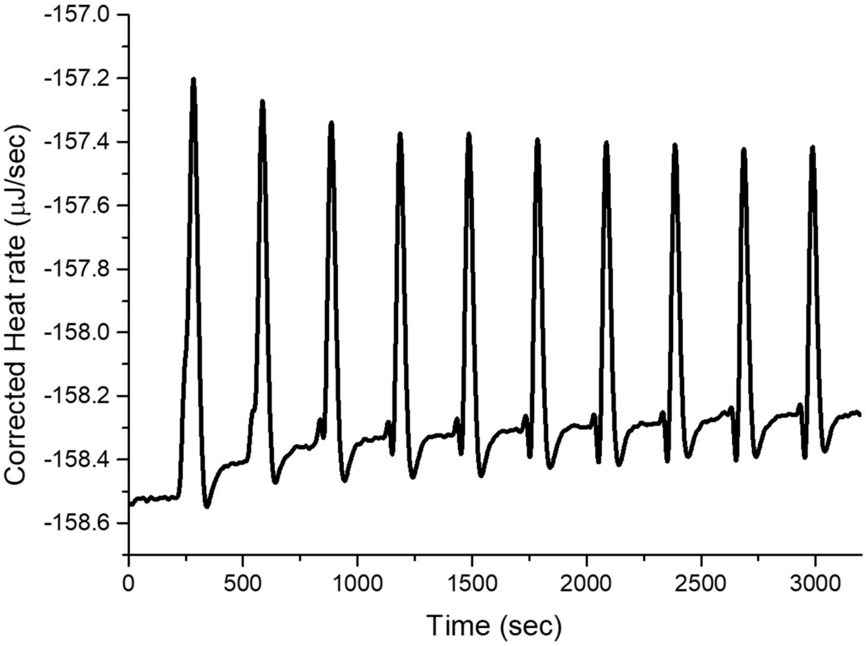


Fig. S6. Raw heat ratio of the PQ binding peptide to DIF measured by ITC.

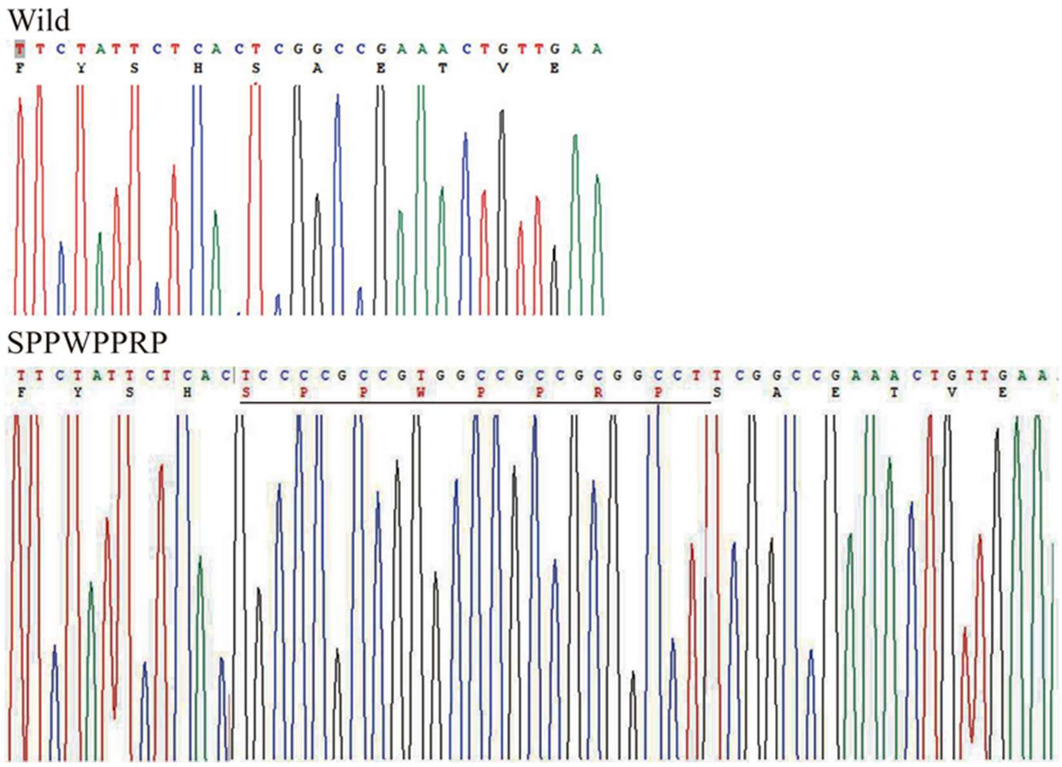


Fig. S7. DNA sequence information of wild-type and the paraquat binding phage measured by high throughput DNA analyser.
